# Supplementary material for: The Long-Term Immunity of a Microneedle Array Patch of a SARS-CoV-2 S1 Protein Subunit Vaccine Irradiated by Gamma Rays in Mice
Source: Vaccines (Basel). 2025 Jan 18;13(1):86. doi: 10.3390/vaccines13010086 (PMC11768753; doi:10.3390/vaccines13010086)
Supplement: Supplementary file 1 [file vaccines-13-00086-s001.zip › vaccines-3339902-supplementary.pdf]

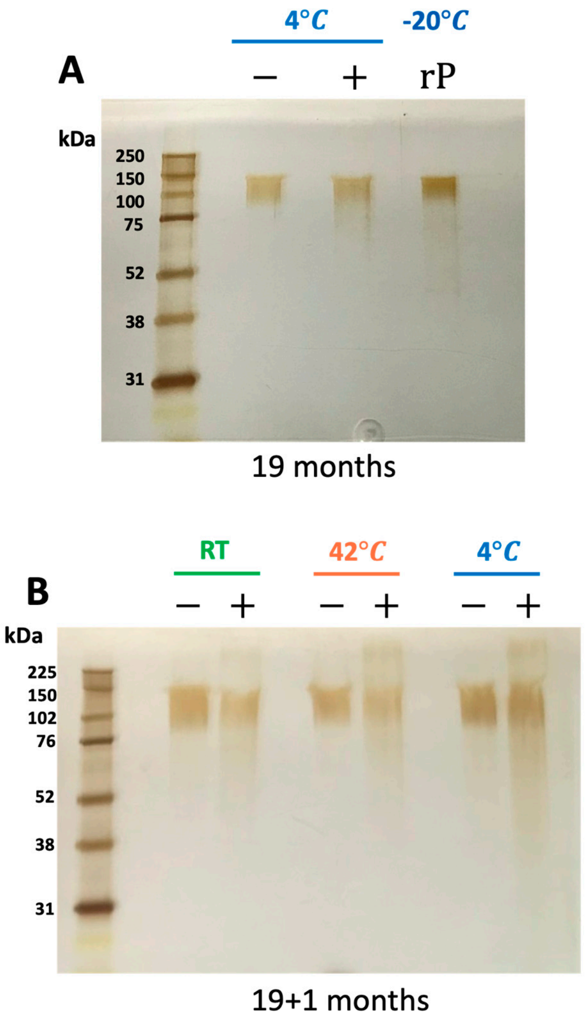

Figure S1. Stability of Irradiated MAP over time (**A**) Silver staining of the recombinant proteins (rP) reconstructed from the non-irradiated (–) and irradiated (+) MAP stored at 4°C for 19 months and recombinant protein (rP) stored at -20°C for 19 months (**B**) rS1RS09 reconstructed from the non-irradiated (–) and irradiated (+) MAP stored for an additional one month at 4°C, RT, and 42°C, after 19 months at 4°C. Approximately 200ng (A) and 300ng (B) of rP were loaded.
